# Supercritical Fluid Extraction of Essential Oil and Sclareol from a Clary Sage Concrete

**DOI:** 10.3390/molecules28093903

**Published:** 2023-05-05

**Authors:** Alessandra Zanotti, Lucia Baldino, Mariarosa Scognamiglio, Ernesto Reverchon

**Affiliations:** Department of Industrial Engineering, University of Salerno, Via Giovanni Paolo II, 132, 84084 Fisciano, Italymrscogna@unisa.it (M.S.); ereverchon@unisa.it (E.R.)

**Keywords:** sclareol, essential oil, supercritical carbon dioxide, multi-step extraction, fractionation

## Abstract

Clary Sage extracts are of industrial interest: in particular, sclareol shows a strong pharmaceutical potential. Supercritical fluid extraction was used to recover compounds of interest from a *Salvia sclarea* L. waxy *n*-hexane extract (“concrete”), using semi-continuous fractionation and a multi-step extraction strategy. Multi-step extraction experiments were carried out in two phases: the first one operated at 90 bar and 50 °C; the second one at 100 bar and 40 °C. GC-MS traces showed that during the first extraction step, only lighter compounds (e.g., monoterpenes, sesquiterpenes, and derivatives) were collected, whereas, in the second step, only sclareol and related compounds were recovered. By adjusting operating conditions (temperature and pressure), selective extraction of different families of compounds was accomplished, with no further need for post-processing of the products. Moreover, using two separators in series, the compounds of interest were fractionated from paraffins and, by changing the operating conditions, the extraction yield increased from about 6.0% to 9.3% *w*/*w* as CO_2_ density increased.

## 1. Introduction

Clary Sage (*Salvia sclarea* L.) is one of the most common herbs cultivated in the Middle East and Southern Europe [1]; in the past few decades, its fields of application have broadened from cosmetics to pharmaceutics, including food packaging, perfumery, and aromatherapy [2,3,4,5,6]. *Salvia sclarea* L. exhibits a strong pharmacological activity, basically related to the composition of its essential oil: terpenoid compounds (i.e., monoterpene hydrocarbons, oxygenated monoterpenes, sesquiterpene hydrocarbons, diterpenes, and oxygenated sesquiterpenes), contained in the volatile fraction of the plant material, are associated with anti-inflammatory, antioxidant, anti-bacterial, and anticancer properties [7,8,9,10,11].

Some attempts to recover compounds of interest from Clary Sage are reported in the scientific literature. Schmiderer et al. [12] obtained both the essential oil (via hydrodistillation) and the dichloromethane extract from several parts of the Clary Sage plant. Essential oil was concentrated in lighter compounds (e.g., linalyl acetate contained up to 63.3 ± 6.7%, and sclareol was limited to 5.9 ± 1.5%), whereas organic solvent extracts contained larger amounts of sclareol (up to 73.6 ± 2.8%). Another comparison between distillation and solvent extraction [13] pointed out that the first process led to low extraction yields (0.55%), whereas solvent extraction (*n*-hexane to produce the “concrete”, and ethyl alcohol to produce the absolute) led to a maximum yield value of 98.3%. Nevertheless, it is imperative to avoid the utilization of organic solvents: they are toxic, polluting, and need expensive post-processing to be separated from the product of interest.

**Figure 1:**
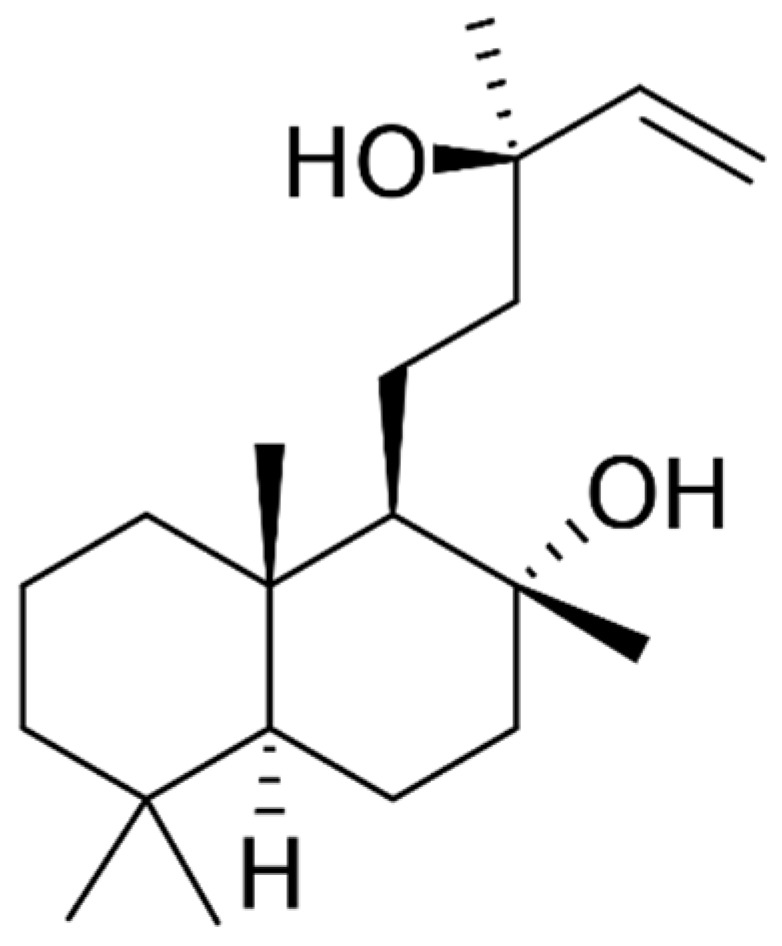
Sclareol chemical structure.

The most relevant compound contained in Clary Sage extracts is sclareol (SCL, Labd-14-ene-8,13-diol, Figure 1), which is a diterpene alcohol (molecular formula C_20_H_36_O_2_). When extracted from the vegetable matter, it is accompanied by several other diterpene alcohols with a similar structure; the most known are cis-abienol and sclareolide, which also present significant pharmaceutical activities [14].

Sclareol is responsible for the antitumor activity of Clary Sage extracts; indeed, it slows down tumor cell line proliferation, inducing apoptosis and DNA damage. It has proven to be effective against multiple cancer types, such as leukemia, breast, colon, lung, cervical cancer, and osteosarcoma [15]. Moreover, its administration induces relaxation and limits hyperglycemia [16,17].

These considerations justify the high industrial attention on these compounds, especially from a pharmaceutical and biomedical perspective. Therefore, it is important to find an extraction process that allows a sclareol-selective recovery, as well as lighter compounds (which still have possible industrial applications). In addition, it is imperative that the extraction process does not rely on the utilization of organic solvents, is eco-friendly, and reduces the need for further post-processing of the extracts.

Supercritical carbon dioxide (SC-CO_2_) is non-toxic, non-pollutant, economic, and can be used at mild temperature conditions. It is recognized as a recommendable extraction fluid, since its properties can be adjusted by properly changing the operating conditions (i.e., pressure and temperature); it shows a gas-like diffusivity and a liquid-like density [18]. Operating in this way, CO_2_ density (and, thus, its solvent power) can be tailored on the specific compounds to recover; therefore, supercritical fluid extraction (SFE) is a more selective process with respect to the other extraction techniques [19,20]. Some attempts to perform SFE to recover compounds of interest from Clary Sage are reported in the literature. Ronyai et al. [21] compared the performance of steam distillation and SFE, operating at 100 bar and 40 °C; they concluded that the most significant difference was related to the amount of sclareol in the extract. The product of steam distillation was deprived of sclareol; instead, the SFE extract was concentrated in sclareol up to 50%. However, some higher-molecular-weight compounds were also detected (i.e., tricosane, tetracosane, and hexatriacontane), reducing the process selectivity towards the compound of interest. Aleksovski et al. [22] attempted CO_2_-assisted extraction from *Salvia sclarea* L., varying operative pressure and temperature. They noted that the extraction yield increased when the CO_2_ density increased, but the process selectivity was negatively affected. Therefore, it can be stated that a successful SFE process relies on two aspects: (i) balance between temperature, pressure, and CO_2_ solvent power; and (ii) separation between compounds of interest (i.e., terpenoids), paraffins, and long-chain alcohols. This balance among operating conditions can be obtained using two separators in series after the extraction vessel; the first one cooled down to 0 °C to drastically reduce the solubility of waxes in SC-CO_2_ [23], and the second one to recover fragrances and sclareol.

To the best of our knowledge, the separation between SCL and sage lighter compounds has not been obtained using SC-CO_2_ extraction. Another limitation of all the applied techniques is the necessity to use very large quantities of the raw vegetable matter. In this work, a different approach is proposed: SFE was applied on a semi-finished material, the so-called “concrete”. A “concrete” is more convenient with respect to the fresh vegetable matter because it occupies smaller volumes, is more concentrated in the compounds of interest, and can be stored at mild conditions [24]. Therefore, the utilization of a sage “concrete” could help to overcome the economic and logistical issues of the supercritical fluid extraction [25,26,27]. For this reason, a supercritical fluid extraction plant used to treat a “concrete” matrix would be much more convenient, since plant volumes are smaller, as well as investment and operative costs.

Therefore, taking into account the advantages related to the utilization of a vegetable “concrete” and the great interest around sclareol-based extracts, the focus of this work consists of the individuation of the correct strategy to perform a selective SC-CO_2_-assisted extraction on a Clary Sage “concrete” to obtain sclareol and related light compounds.

## 2. Results and Discussion

**Figure 2:**
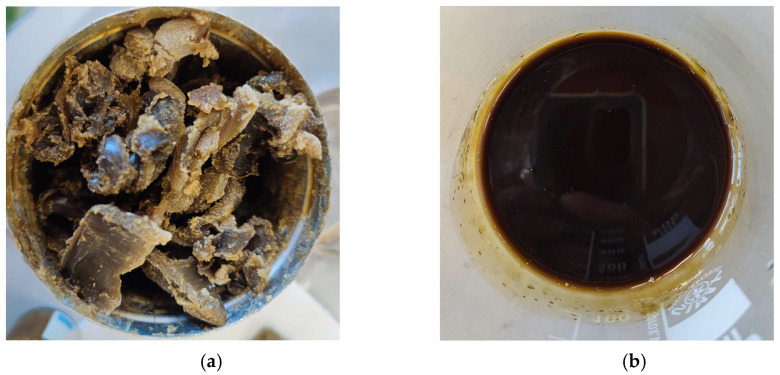
Sage “concrete”: (**a**) as purchased; (**b**) after melting.

Vegetable “concretes” are semi-solid materials that have a waxy consistency and viscosity. Therefore, in order to optimize the contact between the material and SC-CO_2_ in the extraction vessel, sage “concrete” was melted and mixed with 3 mm glass beads. Figure 2 shows the comparison between raw and melted sage “concrete”. In the first case (Figure 2a), the “concrete” was solid, and its consistency could compromise the extraction process, due to a large mass transfer resistance and problems of channeling, whereas in the second case (Figure 2b), after careful mixing with proper packing materials, channeling and caking phenomena could be avoided. The choice of 3 mm glass beads was related to the “concrete” consistency and its exposed surface; smaller spheres could increase the exposed surface, but they would decrease the void degree available to the extraction fluid to pass through the vessel. Indeed, the Clary Sage “concrete” would pack inside the extraction vessel, and SC-CO_2_ could not penetrate inside the vegetable material; extraction would be ineffective and discontinuous, since material depletion would not happen homogenously. On the other hand, larger glass beads would produce smaller pressure drops, and would reduce the risk of channeling and caking, but they would decrease the “concrete” exposed surface and increase the shell thickness on the beads. Therefore, internal mass transfer phenomena could not be neglected, and extraction time would be much longer. Other packing materials (Berl saddles, Raschig rings, etc.) were discarded, since, due to their irregular surface, they did not assure a homogenous distribution of the material on it and in the vessel, compromising the extraction effectiveness. The result of the mixing between glass spheres and Clary Sage “concrete” was instead a system that could be approximated to an inert core (glass) surrounded by an active shell (“concrete”).

As a result of the literature study, multi-stage extraction by SFE was attempted: in the first part of the experiment, the operating conditions were 90 bar and 50 °C, whereas in the second part, they were 100 bar and 40 °C. These operating conditions correspond to a CO_2_ density of 0.29 g/cm^3^ and 0.62 g/cm^3^, respectively. The idea that drove this choice was related to the connection between CO_2_ density and its solvent power: at lower density, the co-extraction of sclareol would have been limited with respect to the recovery of lighter compounds (i.e., monoterpenoids and sesquiterpenoids), or eliminated. Indeed, even though sclareol belongs to the family of terpenoids, being diterpenic, it has a high molecular weight (308.5 g/mol) and, thus, lower affinity with the supercritical fluid [28]. Working in this way, it was expected to extract monoterpenoids and sesquiterpenoids in the first part of the experiment, and mainly sclareol in the second one. In both cases, CO_2_ mass flow rate was set at 0.8 kg/h.

**Figure 3:**
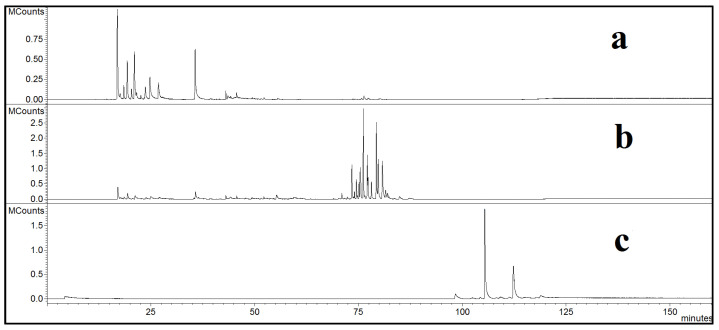
GC-MS traces of: (**a**) extract collected at 90 bar/50 °C; (**b**) extract collected at 100 bar/40 °C; (**c**) waxes.

Figure 3 reports a semi-qualitative representation of the correct choice of operating conditions. In the first step of the extraction (Figure 3a), monoterpenes, oxygenated monoterpenes, sesquiterpenes, and oxygenated sesquiterpenes were collected, whereas in Figure 3b, only sclareol-like compounds can be detected. Indeed, looking both at Figure 3 and the data reported in Table 1, sclareol was extracted when working at 100 bar and 40 °C. Moreover, as evidenced in Figure 3c and Table 2, in the first separator, only paraffinic waxes were collected; this observation confirms that the fractionation system adopted in this work effectively separated compounds of interest from paraffins and long-chain alcohols. In addition, Figure 3a,b and Table 1 show an absence of organic solvent residues; this means that, even if there were some residual traces of *n*-hexane in the starting “concrete” material, they were removed by SC-CO_2_, and extracts were not contaminated for possible pharmaceutical or biomedical applications.

It is worth highlighting that the solution of a multistep extraction promoted the isolation of fragrance compounds from sclareol, avoiding the need for post-processing to separate the latter for high-end applications. This possibility was favored by the utilization of a semi-finished material, such as the “concrete”; in particular, “concretes” are characterized by a homogenous composition, as they are already the product of an extraction step using organic solvents, whereas fresh vegetable materials have an external paraffinic shell, and the compounds of interest are in the internal matrix of the plant [29]. Therefore, in order to penetrate the internal part of the material, a strong mass transfer resistance must be overcome; thus, it is necessary to work at high CO_2_ densities, and fragrance compounds and relatively higher-molecular-weight compounds may be extracted together, reducing the process selectivity.

**Figure 4:**
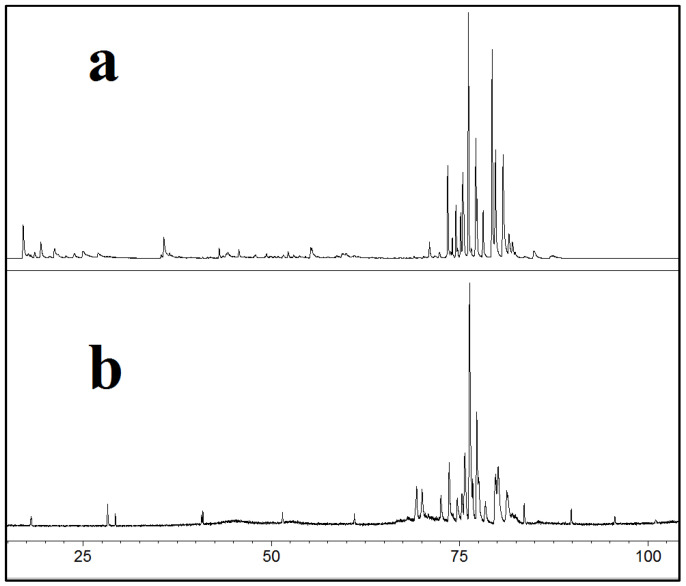
Comparison between: (**a**) extract at 100 bar/40 °C; (**b**) diterpenic reference material.

A comparison of the extract collected at 100 bar and 40 °C and a sclareol reference material (see Materials and Methods) confirmed that the extraction of several diterpenes occurred, since the two GC-MS traces were almost superimposed (Figure 4). As Figure 4b shows, the analytical standard did not show only one peak, but several of them: the highest peak corresponded to sclareol. This means that SCL was the most abundant compound of a compound family with similar molecular structure and molecular weight, whereas the second step of extraction allowed the extraction of the whole family of sclareol.

The results obtained during the first part of this work represent a novelty with respect to the accomplishments reported in the scientific literature. Even though supercritical extraction from Clary Sage was attempted and studied, it never obtained such selectivity. To the best of our knowledge, this is the first time that a multi-step strategy was carried out to separate sclareol from the lighter compounds of the vegetable plant. The results reported in the literature [21,22] show that the main problem was the contamination of the extract by paraffinic waxes co-extracted during the process. The problem of process selectivity was instead overcome in this case, using the strategy of multiple separators in series. In addition, it was possible not only to separate waxes from the extract, but also to separate sclareol from lighter compounds. Even though sclareol (and diterpenoids, in general) have high molecular weights, they have a different chemical structure with respect to paraffinic waxes. For this reason, even when the first separator worked at low temperature, waxes did not contain any trace of sclareol, which could move towards the second separator.

**Figure 5:**
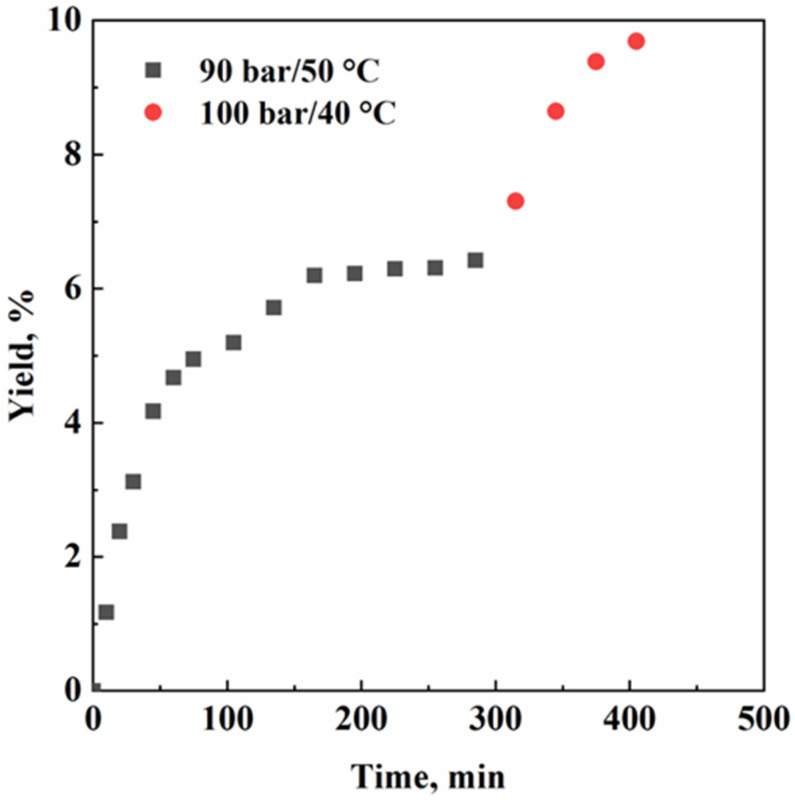
Yield vs. time experimental points.

In the second part of this work, our attention was focused on the effect of the operating conditions on the extraction yield. As evidenced from Figure 5, CO_2_ density immediately affected the extraction yield; the higher the density, the higher the solvent power of the supercritical fluid. The final extraction yield value, after around 7 h of experiment, was equal to 9.3% *w*/*w*.

More specifically, the first part of the process occurred using a lower solvent power of the supercritical fluid; therefore, only compounds with a low molecular weight were extracted. This means that CO_2_ did not have enough solvent power to dissolve every molecule contained in the sage “concrete”. Thermodynamically speaking, during the first extraction step (90 bar/50 °C), the yield value stopped around 6% *w*/*w*; SC-CO_2_ extracted all molecules accessible and soluble in those conditions. Once the first step reached a constant yield value, CO_2_ density was increased, working at 100 bar and 40 °C; in this way, diterpenes could also be solubilized in the supercritical fluid. Therefore, more material was extracted and collected in the second separator. From a macroscopical point of view, this phenomenon caused the increase of the yield value vs. time, which increased up to 9.3% *w*/*w*. These results are coherent with the multi-step extraction approach: the first step is dedicated to more volatile compounds, while the heavier ones do not interact with SC-CO_2_; the second step, instead, starts when the lighter compounds are completely (or almost so) depleted from the starting material, and only sclareol is left to be extracted. From an engineering perspective, higher CO_2_ densities facilitate the diffusion of the extraction fluid into the sage “concrete” shell around the glass beads. In this case, the shell of the vegetable material on the glass beads was extremely thin (around 30 μm; the calculation method is reported elsewhere [24]); thus, internal mass transfer resistances can be neglected. Summarizing, mass transport mechanisms involved in the “concrete” extraction can be associated mainly with external convection and diffusion, when the most appropriate packing material is selected.

## 3. Materials and Methods

### 3.1. Materials and Chemicals

Clary Sage “concrete” was supplied by PIERRE CHAUVET SpA; diterpenic material used as internal standard was purchased from SEPAREX—chimie fine. Prior to mixing with 3 mm glass beads, the “concrete” was melted down to decrease its viscosity and provide a larger exposed surface. The relative amounts of “concrete” and glass beads were calculated by setting the “concrete”/spheres volumetric ratio at 20% *v*/*v*. A total of 15 g of sage “concrete” was used for every extraction experiment. CO_2_ was purchased from Morlando Group Srl (Sant’Antimo, NA, Italy).

### 3.2. Supercritical Fluid Extraction

Extraction and fractionation of Clary Sage “concrete” was performed using a laboratory-scale plant, whose scheme is reported elsewhere [30]. In the present work, a tailor-made extraction vessel (300 cm^3^) was used, coupled with two separators in series. The first separator was cooled down to 0 °C using a refrigerating bath (Julabo, mod. F38); the second separator was set at 35 bar and 60 °C for every extraction experiment to guarantee the transition to the gaseous state of CO_2_ and the collection of the viscous extract. CO_2_ was stored in a liquid–vapor equilibrium in a dedicated tank; then, it was cooled in the refrigerating bath filled with ethylene glycol. Liquid CO_2_ was pumped through the pipelines using a membrane pump (LEWA, mod. Ecoflow), and brought to the desired pressure. The transition to the supercritical condition was accomplished using a thermostatic bath (Julabo, mod. CORIO C-B27) set at the desired temperature. Along the line, after the first separator, a micro-metering valve (Milli-Mite 1300 Series HOKE) was connected to regulate the pressure in the extraction chamber and CO_2_ flow rate. Pressure in the second separator was controlled using a backpressure valve (Tescom, mod. 26-1700); at the end of the line, a rotameter (mod. ASA) and a counter (Sim Brunt, mod. B10) were installed to monitor CO_2_ flow rate continuously. Temperature was controlled in the vessels and along the line using PID controllers (Watlow, mod. 93), heating bands and thermocouples. Pressure was monitored using manometers. Fragrance and sclareol extract were collected in the second separator; by weighing this product at specific intervals of time, global yield (m_extract_/m_concreteloaded_%) vs. time curves were obtained. Waxes, instead, were collected at the end of the experiment, opening the first separator. Extraction experiments were carried out in triplicate to verify the process replicability.

### 3.3. GC-MS Analysis

Analysis and characterization of the extracts and waxes were carried out using a gas chromatography, coupled with mass spectroscopy (GC-MS). In this work, a Varian 3900 apparatus (Varian, Inc., San Fernando, CA, USA), coupled with a fused-silica capillary column (mod. DB-5, J & W, Folsom, CA, USA) was used. It was put together with a Varian Saturn Detector 2100T (Varian, Inc., San Fernando, CA, USA). Helium was the carrier gas, whose flow rate was set at 1 mL/min. Column temperature was set at 40 °C and kept constant for 6 min; then, it was heated at 270 °C, using a 2 °C/min temperature gradient, and held at this temperature for 10 min. Next, 1 μL of a 1:10 *n*-hexane solution was injected in split mode and injector temperature was set at 280 °C. The mass spectrometer was set to work at an ionization voltage of 70 eV in the 40–650 a.m.u. range, and at a scanning speed of 5 scans/s. Once the trace was obtained, the compounds detected were identified by comparison between the literature data and NIST databook. Moreover, a semi-quantitative analysis was carried out, calculating the percentage area under the chromatogram peaks, which corresponds to the relative abundance of compounds in the analyte.

## 4. Conclusions

In this work, the possibility of selectively recovering sclareol from a Clary Sage “concrete” was investigated using a multi-step extraction strategy. By choosing the correct operating conditions (in this case, 90 bar/50 °C and 100 bar/40 °C), the separation of lighter compounds (e.g., monoterpenes and sesquiterpenes) from diterpenes (i.e., sclareol) was performed. The combination between fractionation and multi-step extraction allowed us to obtain uncontaminated extracts, and also to tune their composition. Such technology can be applied for other vegetable matrixes when a precise separation between different species is needed. The utilization of “concretes” played an important role in terms of process time, yield, and selectivity. In future, it could be interesting to investigate the effect of the other operating conditions of the process, as well as the effect of other internal packing types, to improve the extraction process from an engineering point of view. Further studies could be carried out to assess the economic feasibility of the process.

## Figures and Tables

**Table 1:** Area (%) of compounds in the extracts collected in the second separator.

Retention Time, min	Compound	90 bar/50 °C	100 bar/40 °C
16.9	Myrcene	21.1	2.5
18.5	α-terpinene	3	-
19.3	Limonene	8.8	1.6
20.3	β-ocimene, cis	1.9	-
21.0	β-ocimene, trans	14.1	-
23.7	Terpinolene	3.8	-
24.8	Linalool	9.1	-
26.9	α-terpineol	10.1	-
35.7	Linalyl acetate	13.4	2.5
43.1	α-copaene	4.3	-
45.7	Caryophyllene	3.2	-
74.4	Isoabienol	-	9.7
76.2	Sclareol	-	25.3
79.3	Manool	-	15.9
80.8	Thunbergol	-	14.1
85.2	Longifolene	-	3.9

**Table 2:** Area (%) of compounds in the waxes collected in the first separator.

Retention Time, min	Compound	Area (%)
98.2	Eicosane	3.3
105.3	Tricosane	12.0
108.0	Pentacosane	7.5
109.2	Hexacosanol	5.4
111.0	Heptacosane	7.9
119.6	Nonacosane	4.6

## Sample Availability Statement

Samples of the compounds are available from the authors.
